# The Dual Role of Tau in Neuroprotection and Disease: Neuronal Senescence as a Therapeutic Target

**DOI:** 10.1002/alz70855_099490

**Published:** 2025-12-23

**Authors:** Miranda E. Orr

**Affiliations:** ^1^ Washington University School of Medicine, St. Louis, MO, USA

## Abstract

**Background:**

The microtubule‐associated protein tau undergoes reversible post‐translational modifications in response to various stress conditions, preserving neuronal function and stability. However, under sustained stress or pathological conditions, tau may transition from a neuroprotective role to contributing to disease pathogenesis. Cellular senescence exemplifies this duality, with tau inducing neurons to enter a senescent‐like state, termed “neurescence,” which stabilizes stressed neurons but negatively impacts their microenvironment and neuronal circuits.

**Method:**

Spatial proteogenomics was applied to postmortem human brain tissue to investigate tau protein heterogeneity in neurons across the lifespan, identify neurescent cells, and predict therapeutic targets. Pharmacological interventions with senolytics, specifically dasatinib and quercetin (D+Q) or fisetin, were evaluated in preclinical tauopathy mouse models to assess their therapeutic potential. High‐resolution spatial proteogenomic data were analyzed to provide insights into the cell‐type‐specific effects of senolytics. Phase I clinical testing of D+Q in older adults with Alzheimer's disease aimed to determine safety, brain exposure and identify potential treatment‐associated biomarker changes in blood and cerebrospinal fluid.

**Result:**

Spatial proteogenomics highlighted tau's molecular diversity in neurons across the lifespan and identified neurescent cells leading to the identification of potential therapeutic targets. In preclinical models, senolytic treatment with D+Q was superior to fisetin in reducing pathogenic tau. Spatial proteogenomic data provided unprecedented insights into target engagement of each senolytic therapy among brain cell types across multiple regions. Data from preclinical mouse trials aided in the interpretation of fluid biomarker results from human D+Q clinical trials, suggesting that brain‐region‐specific glial populations may be particularly responsive to therapeutic interventions.

**Conclusion:**

Tau‐induced neurescence epitomizes the dual role of tau in both neuroprotection and disease progression. By stabilizing stressed neurons through neurescence, tau prevents immediate neuronal loss but contributes to altered cell states that impair brain function. Targeting tau‐induced senescence with senolytics represents a promising therapeutic strategy, offering critical opportunities to mitigate tau‐driven neurodegeneration and restore brain health. The D+Q combination therapy to clear senescent cells is currently being evaluated in a placebo‐controlled, multi‐site phase 2 trial, SToMP‐AD (NCT04685590).

